# Neural and physiological data from participants listening to affective music

**DOI:** 10.1038/s41597-020-0507-6

**Published:** 2020-06-15

**Authors:** Ian Daly, Nicoletta Nicolaou, Duncan Williams, Faustina Hwang, Alexis Kirke, Eduardo Miranda, Slawomir J. Nasuto

**Affiliations:** 1 Brain-Computer Interfaces and Neural Engineering Laboratory, School of Computer Science and Electronic Engineering, University of Essex, Colchester, CO4 3SQ UK; 2 University of Nicosia Medical School, 21 Ilia Papakyriakou, 2414 Engomi, CY-1700 Nicosia, Cyprus; 3 Centre for Neuroscience and Integrative Brain Research (CENIBRE), University of Nicosia Medical School, 21 Ilia Papakyriakou, 2414 Engomi, CY-1700 Nicosia, Cyprus; 4 Acoustics Research Centre, School of Computing, Science, and Engineering, University of Salford, Manchester, UK; 5 Brain Embodiment Laboratory, Biomedical Engineering Section, School of Biological Sciences, University of Reading, Reading, RG6 6AY UK; 6 Interdisciplinary Centre for Computer Music Research, University of Plymouth, Plymouth, UK

**Keywords:** Emotion, Cognitive neuroscience

## Abstract

Music provides a means of communicating affective meaning. However, the neurological mechanisms by which music induces affect are not fully understood. Our project sought to investigate this through a series of experiments into how humans react to affective musical stimuli and how physiological and neurological signals recorded from those participants change in accordance with self-reported changes in affect. In this paper, the datasets recorded over the course of this project are presented, including details of the musical stimuli, participant reports of their felt changes in affective states as they listened to the music, and concomitant recordings of physiological and neurological activity. We also include non-identifying meta data on our participant populations for purposes of further exploratory analysis. This data provides a large and valuable novel resource for researchers investigating emotion, music, and how they affect our neural and physiological activity.

## Abstract (summary)

**Table:** 

Measurement(s)	brain measurement • response to stimulus • emotion/affect behavior trait • electrodermal activity measurement
Technology Type(s)	electroencephalography (EEG) • electrocardiography • functional magnetic resonance imaging • electrode
Factor Type(s)	age of participants • sex of participants
Sample Characteristic - Organism	Homo sapiens

Machine-accessible metadata file describing the reported data: 10.6084/m9.figshare.12326519

## Background & Summary

Music is an intrinsic part of life for many people. It provides a means to smooth social bonding, a source of entertainment, and provides a sense of identity by communicating, expressing, and inducing a wide range of emotions^1–4^. However, the processes by which music induces emotions are not fully understood^5^. In particular, the links between neurological activity, reported emotions, and music have not yet been explored in sufficient detail to allow a complete theory of the process of emotion-induction by music to be developed^1,6–11^.

Our project, entitled ‘Brain-computer music interfacing for monitoring and inducing affective states’ (http://neuromusic.soc.plymouth.ac.uk/bcmi-midas/index.html), sought to explore relationships between music, emotions, and activity in the brain and body^12–16^. We sought to build relational models describing how these different process relate to one another and to use these models to construct physiologically-driven systems for interacting with music.

Over the course of the project, datasets were collected from a number of different experiments exploring how music affects emotions and related neurological and physiological processes. The studies involved adult participants aged 18 to 66 years old, with approximately the same numbers of males and females. Combinations of music excerpts and computer-generated music were used as stimuli, and participants’ felt emotions were recorded via self-reports as they listened to music via a battery of widely-used methods. Additionally, in a large proportion of the studies neural and physiological data were recorded while participants listened to music. This included, but was not limited to, functional magnetic resonance imaging (fMRI), electroencephalogram (EEG), electrocardiogram (ECG), galvanic skin response (GSR), and respiration rates.

In total our data consists of approximately 150 hours of recordings from 114 participants. The data are summarized in Table 1.

**Table 1:** Summary of datasets.

Name	Recording objective(s)	Type(s) of data	Stimuli	No. of participants	Age range	Hours recording per participant (mean ± STD.)	Stimuli included
Film clips	Identify EEG-correlates of music-induced emotions	EEG, Affective responses (Likert scales)	Film music clips (acoustic)	31 (18 female)	18–66	0.8 ± 4 min	No (available: https://www.jyu.fi/music/coe/materials/emotion/soundtracks/ ^17^).
BCMI calibration	Develop an online brain-computer music interface (BCMI)	EEG, ECG, GSR, Affective responses (FEELTRACE)	Synthetic music	19 (10 female)	19–30	1.2 (±5 min)	Yes
BCMI training	Develop and train an online brain-computer music interface (BCMI)	EEG, ECG, GSR, Affective responses (FEELTRACE)	Synthetic music	10 (7 female)	19–30	3.2 (±32 min)	Yes
BCMI testing	Evaluate an online brain-computer music interface (BCMI)	EEG, ECG, GSR, Affective responses (FEELTRACE)	Synthetic music	8 (6 female)	19–30	1.3 (±16 min)	No (generated during experiment, code provided)
BCMI tempo	Develop and evaluate a BCMI for controlling the tempo of music	EEG	Synthetic music	18 (4 female)	19–28	1 (±5 min)	No (generated during experiment, code provided)
Joint EEG-fMRI music listening	Identify EEG-fMRI correlates of music-induced emotions	fMRI, EEG, ECG, Affective responses (FEELTRACE)	Synthetic music and Classical music clips	21 (10 female)	20–30	1.0 (±5 min)	Yes

## Methods

In this paper we present four key datasets that were recorded during the project and which form the basis of the majority of the research conducted in the project. The four datasets are:

1. The ‘Film clips’ dataset, which contains EEG recorded while participants listened to short music clips extracted from films and chosen to induce specific emotional responses.
2. The ‘Brain-Computer Music Interface’ dataset, which contains EEG and other physiological signals recorded during development and evaluation of the Brain-Computer Music Interface (BCMI) system developed in the project. This dataset is divided into three parts: calibration, training, and testing.
3. The ‘BCMI tempo’ dataset, which contains EEG recorded while developing a BCMI for controlling the tempo of music.
4. The ‘Joint EEG-fMRI’ dataset, which contains EEG and fMRI recorded simultaneously from participants while they listened to both synthetic and classical music and reported their current felt affective states on a continuous basis.

### Film clips

The film clips dataset was recorded as part of a study investigating the effects of short clips of acoustic film music on participants’ emotions and their EEG. Thirty one healthy adult individuals (age range 18–66, 18 female) participated in this study. EEG (approximately 45 mins in total per participant) was recorded from 19 channels positioned according to the international 10/20 system for electrode placement. Participants were asked to listen to a series of short (12 s) clips of music taken from a pre-labeled set of extracts of music from films^17^. They were then asked to report their felt affective states via a series of 8 randomly-ordered Likert questions designed to identify the level of emotional response along 8 axes. These 8 questions allowed the participants to report their music-induced emotions in terms of pleasantness, energy, sadness, anger, tenderness, happiness, fear, and tension.

The experiment consisted of 6 runs of EEG recording. The first and last of these runs were baseline recordings in which participants were instructed to sit still for 300 s and avoid excessive eye movement. The other 4 runs each contained 10 trials, presented in random order to each participant.

Each trial contained a 3 s fixation cross, followed by 12 s of music, a 0.5 s pause and then the 8 Likert questions were presented, in random order, to the participants for them to report on their felt emotions after listening to the music. The specific questions were of the form ‘The music made me feel…’ followed by one of the adjectives: ‘pleasant’, ‘energetic’, ‘tense’, ‘angry’, ‘fearful’, ‘happy’, ‘sad’, or ‘tenderness’. Each question could be answered on a 9-point scale from ‘strongly disagree’ to ‘strongly agree’. Finally, the inter-trial interval was uniformly drawn from the range 2–4 s.

Full details of how the data recorded in this study was processed and analysed, as well as the initial results, are available in^13^. Additional analysis of this dataset, showing how music-induced emotions can be more effectively predicted from combinations of EEG and musical features, are presented in^18^. Finally, this dataset has also been used in the evaluation of a feature selection method^19^.

### Brain-Computer Music Interface (BCMI): calibration, training, testing

A Brain-Computer Music Interface (BCMI) was constructed with the aim of using music as a means of modifying the user’s affective state. Specifically, the BCMI attempted to identify a user’s current affective state and modify it by playing a sequence of generated music pieces in order to place the user in a more desirable affective state (for example by reducing a user’s arousal to calm them down). The BCMI was calibrated, trained, and tested online on a per participant basis, over a number of sessions and days. EEG, GSR, and ECG data from 19 healthy adult participants is available across 3 datasets, corresponding to the calibration, training, and testing sessions (with approximately 1.2, 3.2 and 1.3 hours of recording per participant in each session).

The system was calibrated for each individual user during 1 session, comprising 5 runs during which the user was presented with 40 s music clips targeting different affective trajectories, i.e. shifts from one affective state to another.

During the calibration and training sessions recordings of pre-generated music were used as stimuli. These were generated with the music generator described in^20^ and are included with the datasets made available with this paper. During the testing session the synthetic music was generated in real-time and online (the synthetic generator used to generate the music clips is described in^20^). Because these music stimuli was generated in real time during the experiment they differed between participants and are not included in our datasets. Readers wishing to replicate this experiment may find the details of how to generate the music in^20^. We have also included the code used by this music generator and a short read-me file describing its use with the datasets.

It may also be noted that the same music generator was used to generate the stimuli used in all three stages of the experiment (calibration, training, and testing). Therefore, the stimuli included with the calibration and training datasets may be viewed as representative of the stimuli used during the testing experiments.

The calibration session consisted of 5 runs, each of which contained eighteen trials in which participants listened to music and reported their current felt emotions as they listened to the music. Each trial began with a fixation cross, which was displayed for 1–2 s. This was followed by a 21 s music listening and reporting period, in which participants both listened to a randomly selected piece of pre-generated music and reported their current felt emotions via the FEELTRACE interface^21^. This was followed by two self-assessment manikins, which were used to allow the participants to report their current felt valence and arousal. Finally, a brief distraction task was used to minimise the effect of sequential presentations of different pieces of music. Participants were asked to count the number of high-pitched beeps they heard in a 15 s period and then rest for 2.5 s.

Following calibration, the BCMI system was trained for each individual user over a number of days, during which participants listened to 40-s synthetic music clips targeting two affective states, as defined by valence and arousal (the first 20 s targeted one affective state, then the second 20 s targeted a different affective state). During each 40 s music listening period participants reported their current affective states via the FEELTRACE interface. All other details of the trial structure were the same as the calibration session.

Data were recorded over 3 sessions (conducted over separate days), each containing 4 runs (same day) of 18 music-listening trials each. The training sessions served a twofold purpose; firstly, to allow the system to learn how to modify the music generator’s parameters in order to achieve a desired affective trajectory (i.e. shift from one affective state to another) for each individual user; and, secondly, to identify neural signatures of affect and how these varied for different affective states.

The system was then tested in a fully online and real-time testing session, during which the system performance in inducing selected affective trajectories for each individual user was investigated. The data recorded during this session is contained in the BCMI-testing dataset.

The testing session had the same session and trial structure as the training session. Each participant completed one testing session, which contained 4 runs, with 18 trials in each run. Each trial contained a 60 s period during which music was played. In the first 20 s a specific affective state was targeted by the music. The next 20 s were used to attempt to identify the user’s current affective state. Finally, the last 20 s were used by the BCMI to attempt to dynamically alter the user's affective state.

The data recording methods and the BCMI system are described in full in^22^.

### Brain-Computer Music Interface (BCMI) tempo

An additional BCMI system was also developed to allow users to control the tempo of pieces of music via active modulation of their motor imagery. Specifically, the BCMI was developed to allow users to increase the tempo of a piece of music by concentrating on kinaesthetic imagination of upper limb movement. Music was generated dynamically during the experiments via a music generator developed in the project and described in^20^. The tempo of the music could be reduced by relaxing and increased by kinaesthetic motor imagery.

EEG was recorded from 19 channels positioned according to the international 10/20 system for EEG electrode placement. Recording was made via a BrainProducts BrainAmp system at a sampling rate of 1,000 Hz with impedances below 5 kΩ.

Feedback to the users took three forms: visual (via an onscreen ball), auditory (via the music), and a combination of visual and auditory. The data comprises approximately 1 hour of EEG recordings per participant.

This 1 hour recording session was split into 9 runs, the first of which was a calibration run, with the remaining 8 runs used to train the user to control the BCMI. The calibration run was split into 30 trials, while each subsequent run was split into 18 trials.

Each trial consisted of a fixation cross displayed on screen for 4 s. This was followed by 12 s in which participants were instructed to attempt to use kinasthetic dominant hand motor imagery to attempt one of the following tasks: move a ball either to the top of the screen or to the bottom, increase or decrease the tempo of a piece of music, or simultaneously attempt both tasks. This was followed by feedback (a smiling or frowning face depending on whether the user succeeded in the cued task or not), which was displayed on screen for 0.5 s.

Full details of the dataset are provided in^15^. Additionally, further analysis of the data was conducted to show how changes in the tempo of the music acted to entrain changes in event-related (de)synchronization (ERD/S) in the EEG^23^.

### Joint EEG-fMRI

A final dataset was recorded via a joint EEG-fMRI imaging modality from a cohort of 21 healthy adult participants (20–30 years old, all right handed, 10 female) while they listened to music. Two sets of stimuli were used: the first comprised synthetic music generated with the intention of inducing a wide range of different affective states, while the second set comprised a small set of classical music clips chosen for their ability to induce a wide range of different affective states.

Participants reported their current felt affective states on a continuous basis via the FEELTRACE interface^21^ while they listened to the different music clips. Additionally, control conditions were used to control for the arm and eye movements associated with the use of the FEELTRACE interface.

EEG was recorded via 31 channels (with 1 additional channel used for ECG recording) via an MRI-compatible BrainAmp MR system at a rate of 5,000 Hz with impedances below 10 kΩ. EEG channels were placed according to the international 10/20 system for EEG electrode placement.

fMRI recordings were made via a 3.0 Tesla Siemens Magnetom Trio scanner with a 37-channel head coil. Anatomical scans were first made from each participant (field of view = 256 × 256 × 176 voxels, TR = 2020 ms). This was followed by functional sequences (TR = 2000 ms, echo time = 30 ms, field of view = 64 × 64 × 37).

The paradigm consisted of three 10-minute runs during which participants listened to randomly-selected pieces of generated music, followed by a 2 minute n-back task, and then a 30 minute run in which they listened to a set of classical music pieces. During each music-listening trial participants reported their current felt emotions via the FEELTRACE interface.

Each trial began upon a transistor-transistor logic pulse from the fMRI scanner. This was followed by a fixation cross for 1–3 s, followed by a music listening and reporting period, which lasted for 40 s for the generated music and for a variable length of time for the classical music listening session (the length was dependent on the length of the piece of classical music and varied between 2–3 minutes). This was followed by a 0.5 s inter-stimulus interval before waiting for the next TTL pulse from the fMRI scanner to start the next trial.

Full details of this dataset, its analysis, and the experiment design are provided in our associated publication^16^.

### Quality assurance (QA)

All physiological data was visually inspected for presence of artefacts by researchers with at least 5 years experience at the time of inspection. In all cases the inspector was blinded to the associated labels within the data (stimuli used, affective responses reported by participants etc.).

Artefact contaminated trials were corrected or removed in our analysis of the datasets, as described in the associated publications. However, the data are published here in its original, uncorrected, form to allow maximum flexibility in its use.

For reference the artefact removal process and proportion of artefact contaminated trials removed from each dataset in our original publications are described in Table 2.

**Table 2:** Summary of artefact removal methods applied in our analysis of the datasets.

Dataset	Artefact removal method	Proportion of trials removed
Film clips	ICA, followed by visual inspection of individual ICs by a blinded reviewer, followed by removal of trials rated as containing EMG, movement, failed electrode artefacts, or amplitudes >±100 *μ*V	31.03%
BCMI calibration	Automated removal via the approach described in^33^, followed by amplitude threshold-based rejection (±100 *μ*V) and visual spot-checking.	Approx. 10%
BCMI training	Automated removal via the approach described in^33^, followed by amplitude threshold-based rejection (±100 *μ*V) and visual spot-checking.	Approx. 10%
BCMI testing	Post-hoc amplitude thresholding (±100 *μ*V) and visual spot-checking.	8.95%
BCMI tempo	Visual inspection by a blinded reviewer. Trials were rejected if they contained artefacts on channels F3, T3, C3, Cz, or P3 during control periods.	12.36%
Joint EEG-fMRI music listening	Automated artefact removal via the approach described in^24^ followed by ICA, rating of ICs by a blinded reviewer, and manual removal of components.	8.9 components (out of 31) removed.

Note, the original data is provided with this paper and contains artefacts.

**Table 3:** Summary of code scripts.

Script	Related dataset(s)	Description
load_BCMItempo.m	BCMItempo	Load data recorded during experiments to train and evaluate the tempo-based BCMI.
load_filmClips.m	Film clips	Load data recorded during the film clips experiments.
load_phBCMIcalibration.m	BCMI calibration	Load data recorded during the calibration sessions of the affective BCMI experiments.
load_phBCMItraining.m	BCMI training	Load data recorded during the training sessions of the affective BCMI experiments.
load_phBCMItesting.m	BCMI testing	Load data recorded during the testing sessions of the affective BCMI experiments.

EEG data was recorded with minimal impedance on all channels using CE certified research grade EEG measurement systems. All experiments were conducted by, or with supervision from, researchers with a minimum of 5 years experience conducting EEG and physiological measurement studies.

All EEG data were recorded via the standard or extended 10/20 international systems for EEG electrode placement. However, the numbers and locations of recording sites differ across studies according to the particular needs of each study.

The fMRI data has been pre-processed to remove face information. Specifically, the anatomical scans were processed to remove the faces of the participants (note this was not necessary on the functional scans as the field of the functional scans only covered the brain and some parts of the skull). The data are, otherwise, provided in raw (unprocessed) format along with associated anatomical scans for each participant. This is to allow maximum flexibility in re-use of the data.

The EEG recordings included in this dataset have already been pre-processed to remove fMRI scanner noise via the average artefact substract (AAS) method^24^. The EEG has also been co-registered to the fMRI recordings. Specifically, the first TTL trigger recorded in the EEG dataset (see the event description file) corresponds to the time point of the first scan image in the fMRI dataset (functional scan set).

### Ethics

All the studies described in this paper were reviewed according to the procedures of the University of Reading research ethics committee and given favourable opinion for conduct. All experiments were performed in accordance with all relevant guidelines and regulations. Informed written consent was obtained from all participants in all sets of experiments.

## Data Records

All data are available on the OpenNeuro data archive. The Film Clips dataset can be accessed from the Open Neuro archive via 10.18112/openneuro.ds002721.v1.0.1. The data are provided in BIDS format^25,26^, with each folder corresponding to data from one participant^27^.

The BCMI system data are provided in 3 separate datasets, corresponding to the calibration, training, and testing phases of the BCMI system. Even though the number of participants varies between datasets, participant codes are the same across all three datasets for consistency. The BCMI calibration dataset can be accessed from the Open Neuro data archive via 10.18112/openneuro.ds002722.v1.0.1^28^. The BCMI training dataset can be accessed via 10.18112/openneuro.ds002724.v1.0.1^29^. The testing dataset can be accessed via 10.18112/openneuro.ds002723.v1.1.0^30^. The data are provided in BIDS format, with each folder corresponding to data from one session for each participant.

The BCMI tempo dataset can be accessed from the Open Neuro Data Archive via 10.18112/openneuro.ds002720.v1.0.1^31^. The data are provided in BIDS format, with each folder corresponding to data from one participant.

The joint EEG-fMRI dataset can also be accessed from the Open Neuro data archive via 10.18112/openneuro.ds002725.v1.0.0^32^. The data are provided in the BIDS format.

## Technical Validation

We performed technical validation for each dataset individually as a part of the initial analysis we performed after each experiment.

Specifically, for the ‘Film clips’ dataset we first examined the participant response data to ensure it contained values within the expected numerical ranges and at the correct points in time. We then segmented the EEG according to the recorded meta data before visually inspecting it to ensure it was reasonably clean of artefacts and physiologically meaningful. This was done by a researcher with over 8 years of experience of EEG inspection (author ID). Finally, the segmented EEG was used to measure changes in prefrontal asymmetry for trials in which participants reported different levels of felt emotions. The results were then compared to those expected from literature. The full details are reported in^13^.

For the ‘BCMI’ datasets (‘calibration’, ‘training’, and ‘testing’) we technically validated the data via a similar process. The time series of participant responses (recorded via the FEELTRACE interface) were first visually inspected to ensure they were recorded at the correct times and fell within expected numerical ranges. The EEG and other physiological signals were then visually inspected by experienced researchers (authors ID and DW) before segmenting them into trials and plotting the resulting changes in prefrontal asymmetry against the participant’s reports of their felt emotions. This allowed us to validate that the EEG contained expected neural correlates of affective responses to the stimuli. Full details are reported in^22^.

For the ‘BCMI tempo’ dataset technical validation was performed after completion of the online BCI control experiment. Specifically, the EEG was visually inspected by an experienced researcher (author ID) to ensure it contained a reasonable level of noise and conformed to physiological expectations. The details are reported in^15^.

Finally, technical validation of the joint EEG-fMRI dataset was performed in a multi-step process. First the triggers, transmitted from the fMRI scanner to the EEG recording, were checked to ensure they occurred at expected times (once every 2 s) and could be used to co-register the EEG and fMRI recordings together. The fMRI data were manually pre-processed using the Statistical Parametric Mapping (SPM version 12) toolbox in Matlab and visually inspected at each stage to ensure correct recording of all parts of the data, including structural (anatomical) scans and functional scans. The associated EEG data were cleaned to remove fMRI scanner artefacts using the average artifact subtraction method^24^ implemented in the ‘BrainVision Analyser’ software (BrainProducts). The resulting EEG was then visually inspected by an experienced researcher (author ID) to ensure it was reasonably clear of artefacts and conformed to physiological expectations. Full details are provided in^16^.

## Usage Notes

The data from all the datasets are saved via the BIDS format^25,26^. Readme files are included with each dataset to describe the particular structure of the data.

## Data Availability

We have prepared scripts to demonstrate how to load the datasets into Matlab ready to be pre-processed. These scripts are listed and introduced in Table 3. All scripts are placed within the sub-folder ‘code’ in the respective BIDS formatted datasets. Interested readers are also directed to the BIDS Matlab repository, which provides tools for working with BIDS-structured data in Matlab (https://github.com/bids-standard/bids-matlab). We have also provided the code used by the music generator in the experiments that generated the ‘BCMI tempo’ and ‘BCMI testing’ data-sets. This is located in the ‘code’ sub-folder in each dataset along with a document describing the music generator. Please note, the music generator is not a single piece of code, but rather a collection of scripts and functions that work together with some proprietary software to generate the music. The code was written in Max/MSP version 6.1 and this software will be needed to get the music generator to work. Further details are also provided in^20^.
